# The impact of National Health Insurance upon accessibility of health services and financial protection from catastrophic health expenditure: a case study of Savannakhet province, the Lao People’s Democratic Republic

**DOI:** 10.1186/s12961-019-0493-3

**Published:** 2019-12-16

**Authors:** Somdeth Bodhisane, Sathirakorn Pongpanich

**Affiliations:** College of Public Health Science (CPHS), Chulalongkorn University, Institute building 3 (10th–11th floor), Chulalongkorn soi 62, Phyathai Rd, Bangkok, 10330 Thailand

**Keywords:** Accessibility to health services, National Health Insurance, financial protection, Community-Based Health Insurance, Laos People’s Democratic Republic, health services, hospitalisation, catastrophic health expenditure, health policy

## Abstract

**Introduction:**

Many schemes have been implemented by the government of the Lao People’s Democratic Republic to provide equity in health service utilisation. Initially, health service utilisations were fully supported by the government and were subsequently followed by the Revolving Drug Fund. In the 2000s, four health financing schemes, namely the Social Security Organization, the State Authority for Social Security, the Health Equity Fund and Community-Based Health Insurance (CBHI), were introduced with various target groups. However, as these voluntary schemes have suffered from a very low enrolment rate, the government decided to pilot the National Health Insurance (NHI) scheme, which offers a flat, co-payment system for health service utilisation. This study aims to assess the effectiveness of the NHI in terms of its accessibility and in providing financial protection from catastrophic health expenditure.

**Methods:**

The data collection process was implemented in hospitals of two districts of Savannakhet province. A structured questionnaire was used to retrieve all required information from 342 households; the information comprised of the socioeconomics of the household, accessibility to health services and financial payment for both outpatient and inpatient department services. Binary logistic regression models were used to discover the impact of NHI in terms of accessibility and financial protection. The impact of NHI was then compared with the outcomes of the preceding, voluntary CBHI scheme, which had been the subject of earlier studies.

**Results:**

Under the NHI, it was found that married respondents, large households and the level of income significantly increased the probability of accessibility to health service utilisation. Most importantly, NHI significantly improved accessibility for the poorest income quantile. In terms of financial protection, households with an existing chronic condition had a significantly higher chance of suffering financial catastrophe when compared to households with healthy members. As probability of catastrophic expenditure was not affected by income level, it was indicated that NHI is able to provide equity in financial protection.

**Conclusion:**

The models found that the NHI significantly enhances accessibility for poor income households, improving health service distribution and accessibility for the various income levels when compared to the CBHI coverage. Additionally, it was also found that NHI had enhanced financial protection since its introduction. However, the NHI policy requires a dramatically high level of government subsidy; therefore, there its long-term sustainability remains to be determined.

## Introduction

The cost of obtaining and accessing proper healthcare in developing countries is relatively higher when compared to richer and more developed countries due to the prevalence of fees or health service charges combined with the high transportation costs encountered by people who have to travel long distances for treatment; these may include both medical and non-medical expenditures. Medical expenditure refers to direct payment paid to health facilities, whereas non-medical expenditure refers to other costs that may occur while receiving health services, including transportation costs, loss of opportunity cost from being unproductive, etc. Substantial levels of healthcare expenditure could lead patients and/or their family into financial catastrophe and impoverishment. Conversely, untreated illness could also push them into poverty through loss of productivity during such times. Domestic financial resources are unevenly distributed when providing for the needs of the poorer sectors of the population, leading to it taking a considerable amount of time to obtain funding from donors or to arrange loans from external sources. Developing countries are unable to collect significant amounts of tax revenue, face insufficient and volatile external funds, suffer from high costs of accessing healthcare services, have inequalities in the health services provided, lack service-minded health practitioners, have inefficient financial management and transparency issues, demonstrate limited accountability in their financing system, and lack scientific evidence for priority policy setting [1].

National Health Insurance (NHI) schemes have been initiated in more than 60 developing countries. In Africa, Tanzania’s National Health Insurance Fund was established in 2001, while Ghana’s NHI was promulgated in 2003 [2]. In southeast Asia, Cambodia’s Health Equity Fund was piloted in 2000, whereby most of the financial resources were supported by donors in order to compensate for poor people’s medical expenditure [3]. After the financial crisis in 1997, the Indonesian government established a tax-financed scheme targeting poor people, where health providers are paid on a case mix-adjusted basis [4]. In 1995, the Philippine Health Insurance Corporation (PhilHealth) was created with the aim to establish universal health coverage in the Philippines; it is a government owned and controlled corporation with tax exemption [5].

Inadequate accessibility to quality healthcare for poor households is considered an important issue for both low- and middle-income countries. These countries have acknowledged and highlighted the existing gap in accessibility and governments need to develop effective strategies to improve equity [6]. It has been estimated that 1.3 billion people around the world are unable to access affordable and effective healthcare. For households with access, approximately 170 million people have been forced to spend more than 40% of their household income on medical treatment, which forces them into financial catastrophe [7]. Financial catastrophe refers to the situation where patients are required to pay or co-pay for their health care and that expenditure is greater than, or equal to, 40% of the non-subsistence income within the household [8].

The government of the Lao People’s Democratic Republic has seen the importance of health service utilisation associated with health financing. Despite an increase in the national healthcare budget to 6%, the figure remains low in comparison to other countries in the region [9]. The health sector in the Lao People’s Democratic Republic is financed through three major sources, namely out-of-pocket (OOP) expenditure (covering 48% of total health expenditure), non-government organisations or donors (covering 32%), and the final 20% is covered by the government budget (allocated by the Ministry of Finance). The contribution from the government budget could be raised to 36% if grant aid was included [10].

### The setting and overview of health financing in the Lao People’s Democratic Republic

The Lao People’s Democratic Republic is a country in the South East Asia region, known for its resources and rich biodiversity. It has a population of 6.492 million and has rapidly advanced after transforming from a centrally planned economy to a market-oriented economy in the 1980s. The rural population still accounts for approximately 61% of the total population, many of whom are engaged in the agricultural sector and the majority earning a low income [11]. Following the establishment of the Lao People’s Democratic Republic, health service utilisation was financed through government budget, with most of the financial resources supported by the People’s Republic of China, the Soviet Union and Vietnam. At that time, a limited range of health services were available ‘free of charge’. Although small, it was an improvement of the government facility network. In the latter years, there has been a decline in financial support from the traditional partners; consequently, this has forced an increased reliance on OOP (household) and community support for healthcare expenditure [12].

As the Lao People’s Democratic Republic transformed from being a controlled to a market-oriented economy in 1986, the Revolving Drug Fund (RDF) was initiated as part of its community health programme. The fund received financial support from non-governmental organisations in the 1990s. In 1993, a national drug policy was approved in order to enhance the accessibility and affordability of essential medicines [13]. A previous study showed that the RDF covered 44% of health facilities, 62% of district hospitals, 94% of provincial hospitals and 6% of villages, with the RDFs being operated by different organisations in various settings [14]. The RDFs were able to ensure systematic financial management and the availability of essential drugs in public hospitals. Under this policy, the RDFs were the major means of financial resources for public hospitals, generating a situation where health practitioners over-prescribed and readily responded to requests from patients for unnecessary drugs. Drug procurement was not adequately monitored and controlled, and often accounted for more than 40% of the budget. Hence, drug expenditure may have been as high as 80% of the total health-related expenditure of a health facility [15].

The government has acknowledged the importance of health service utilisation associated with health financing. The health sector of the Lao People’s Democratic Republic is financed through three major sources, namely OOP expenditure covering 48% of total health expenditure, non-government organisations, or donors and government budget [10]. In order to provide both financial protection and accessibility to health services, four health financing schemes have been introduced to various target groups of the population — (1) the State Authority for Social Security, a mandatory scheme for government officials; (2) the Social Security Organization (SSO), for private-sector employees (a contributory, payroll-financed scheme); (3) the Health Equity Fund (HEF) developed for the poor, generally financed by external donors; and (4) Community-Based Health Insurance (CBHI), a voluntary scheme for non-poor independent workers and those in the informal sector (a fully contributory scheme) [16]. However, the outcomes of these heath financing schemes still fall far short of the expectations, particularly the CBHI scheme. Despite offering both inpatient department (IPD) and outpatient department (OPD) coverage, benefit packages remained inadequate and inefficient, constrained by the low capitation fee set by the government. Additionally, the mandatory SSO enrolment has been weakly enforced since the introduction of the scheme, many employers have cancelled their membership of the SSO and many state-owned enterprises as well as private employers that should have enrolled in the SSO have mostly failed to do so. As a voluntary scheme, the CBHI merely covers 12% of its 3.21 million target population. The HEF scheme was exclusively developed for poor households, covering 192,000 of the population or 12% of its target. The scheme is largely financed or reliant on grant aid, being considered as unsustainable and inefficient in the long run. Due to high implementation costs, there are inadequate financial resources available to subsidise the poor households being exempt from paying user fees; the costs are sometimes covered by the hospital’s own revenue, which discourages the hospitals and health personnel from providing health services to poor households [10, 17].

### National Health Insurance (NHI)

In recent years, the Lao People’s Democratic Republic has made acceptable progress in developing its healthcare system. However, the percentage of total health expenditure, with respect to GDP, was only 2.81%, which is still less than neighbouring Greater Mekong Subregion countries such as Thailand (3.77%), Myanmar (4.95%), Vietnam (5.65%), Cambodia (5.98%) and China (5.32%) [18].

Regardless of the recent reinforcement of public health financing, the Lao People’s Democratic Republic is still categorised as being inconsistent and low level in its government spending. OOP spending is considered as a major means of financing healthcare provision, which deters poor households from using health services and even pushes them into poverty due to unaffordable costs. Moreover, the Lao People’s Democratic Republic also relies on health spending from external sources rather than the income level of the Lao People’s Democratic Republic. In order to achieve universal health coverage, the government of the Lao People’s Democratic Republic needs to inject financial resources into the health system, especially from domestic revenue sources, and to minimise the reliance on OOP spending [9]. As part of the implementation of its 2030 agenda for sustainable development, the government aims to expand the accessibility of health services for its population, especially the elderly and people with disabilities [19]. In 2017, the government of the Lao People’s Democratic Republic allocated a budget of 180 billion LAK (approximately 20 million USD) to develop NHI through a combination of three healthcare schemes (SSO, CBHI and HEF) that can potentially cover 75% of the total population. Similar to its predecessor, NHI offers both OPD and IPD services, where the contribution rates depend on the location of the health service provided. As shown in Table 1, OPD patients are expected to pay a flat contribution rate of 5000 LAK (0.60 USD) at a village health centre, 10,000 LAK (1.20 USD) at a referral hospital and 15,000 LAK (1.80 USD) when using provincial hospitals. IPD services are only available in district hospitals and referral/provincial hospitals, where patients are expected to pay a flat contribution rate of 30,000 LAK (3.60 USD). In the case of patients transferred from OPD to IPD, they are required to pay an additional amount of 20,000 LAK (2.40 USD) and 15,000 LAK (1.80 USD) in district hospitals and referral/provincial hospitals, respectively. However, patients are also expected to pay 25% (as a co-payment) for surgery or treatment that costs over 5 million LAK (600 USD) [20–22]. District hospitals, such as Champhone district hospital, are unable to handle orthopaedic and brain surgery, and therefore most of these patients are directed to visit a referral hospital. Additionally, if necessary, patients staying in district hospitals for more than 3 days are advised to transfer to a referral hospital.

**Table 1:** NHI contribution system

Health facilities	OPD contribution	IPD contribution	Both OPD and IPD^a^
Village health centre	5000 LAK (US$0.60)	N/A	N/A
District hospital	10,000 LAK (US$1.20)	30,000 LAK (US$3.60)	30,000 LAK (10,000 + 20,000) LAK^b^ (US$3.60)
Referral/provincial hospital	15,000 LAK ($US1.80)	30,000 LAK (US$3.60)	30,000 LAK (15,000 + 15,000) LAK^c^ (US$3.60)

*IPD* inpatient department, *OPD* outpatient department

^a^Transfer from OPD to IPD

^b^At district hospital, patients already paid 10,000 LAK ($1.20) for OPD, if they are transferred to IPD they need to pay an additional 20,000 LAK ($2.40), which amounts to a total of 30,000 LAK ($3.60)

^c^At referral/provincial hospital, patients already paid 15,000 LAK ($1.80) for OPD, if they are transferred to IPD they need to pay an additional 15,000 LAK ($1.80), which amounts to a total of 30,000 LAK ($3.60)

Particularly, the NHI was piloted in Savannakhet province (in August 2017) with the ultimate goal of enhancing the accessibility of quality/reliable health services [23]. There has been no scientific evidence, or research conducted, to find out the effectiveness of NHI; the main objective of this study is to assess the impact of NHI in providing accessibility to public hospitals and in offering financial protection from catastrophic expenditure related to health service utilisation, when compared to the proceeding CBHI scheme.

## Methodology

### Research design

This study applies a cross-sectional method to assess the impact of NHI in terms of accessibility to health service utilisation and in providing financial protection against catastrophic health expenditure. The Andersen Behavioral Model, comprising of predisposing, enabling and needs-based characteristics are used as guidelines to identify the factors that directly, and indirectly, impact on both accessibility and financial protection from catastrophic expenditure [24, 25], was used. The outcome was then used to compare with the results of earlier researches to find out the effectiveness of the CBHI scheme, which was the previous scheme that covered the highest possible enrolment numbers [26, 27].

### Data collection

The data collection process was implemented in hospitals during September to November, 2018, in Savannakhet province. The data collection was implemented in two hospitals — Savannakhet Provincial Hospital, a referral hospital in Kaysone Phomvihane district, and Champhone District Hospital in Champhone district, where the total sampling number of 342 was equally divided into two groups, each of 171 households. The settings were identical to the previous study in order to be comparable with the CBHI scheme and the sample size was based on a proportion of the latest study on the effectiveness of the CBHI scheme [27]. A structured questionnaire was used to retrieve all required information, including information on household socioeconomics, accessibility to health services, and financial payment for both OPD and IPD services. Heads of household, being permanent residents (residing more than 6 months) in Kaysone Phomvihane and Champhone districts, were eligible to answer the questionnaire as their households’ representative. The data collection process was implemented at the OPD of the provincial hospital (referral hospital) in Kaysone Phomvihane district and Champhone district hospital (district hospital). These hospitals were selected to compare the results of this study with those of previous studies of the CBHI scheme, for which respondents were selected by a systematic random sampling technique. At the OPD section, patients need to register (in the waiting list), this study systematically selected those patients from the waiting listlist. The interview session before or after receiving health services. During the data collection process, 14 and 10 respondents refused to participate in Kaysone Phomvihane and Champhone districts, respectively, with most stating that they did not have the time or were not interested in the health financing system or in the research topic.

### Data analysis and interpretation

Both descriptive and inferential statistical techniques were used in the analysis of effectiveness, in terms of enhancing accessibility and financial protection, of the NHI. The descriptive data presents the sociodemographics of all respondents and their households. Inferential statistical analysis includes two binary logistic regression models; the first binary logistic regression model was used to analyse the probability of health service utilisation (as a proxy to accessibility to healthcare services). Regarding the assumption that probability of hospitalisation had been used as the proxy to accessibility to health services, this assumption possibly creates bias as these interviews were conducted in hospitals; all respondents would report receiving OPD health services (at the time of conducting the interview). However, the time at the hospital during the interview was not counted; we attempted to minimise this bias by tracing back 3 months for OPD and 1 year for IPD; all 342 households were taken into this model (finding the probability of hospitalisation under NHI). Independent variables, based on the demand and supply sides of the health system, were considered, including gender (of respondent), marital status (of respondent), age (of respondent), occupation (of respondent), level of education (of respondent), size of household (of household), level of income (of household), closest health facilities (of household), travel time to health facility, district of residence, OPD use within 3 months, and IPD use within 12 months. Conversely, the dependent variables were health service utilisation (as a proxy of accessibility to healthcare services) and financial protection against catastrophic expenditure.

The second logistic regression model was used to analyse the probability of financial catastrophe after the NHI’s establishment. Similar to the first model, independent variables were also based on the Andersen Behavioral Model, whereas the dependent variable was the probability of the household suffering from financial catastrophe. Consequently, only 106 households, who reported they had used the health IPD service within the previous 12 months, were used in this model.

### Validity

Content validity was deliberately checked with the assistance of an expert from the College of Public Health Science, Chulalongkorn University, to ensure that the tool (structured questionnaire) covered all the information required. Moreover, construct validity was also used in order to ensure that the theoretical foundation supports the conceptual framework, which includes multiple sources of information, key informant reviews and establishing the chain of evidence [28].

### Funding

This study has been supported by funding from Rachadapisaek Sompot Fund of the Graduate School, Chulalongkorn University.

### Ethics

The authors obtained ethical approval from National Ethic Committee for Health Research (NECHR), National Institute of Public Health, Lao People’s Democratic Republic. The authors obtained ethical approval from the National Ethic Committee for Health Research (NECHR), National Institute of Public Health, Lao People’s Democratic Republic.

## Results

Tables 2 and 3 illustrate the descriptive statistics between the sociodemographic of respondents/households in relation to hospital admission and catastrophic health expenditure from 342 households collected in both Kaysone Phomvihane and Champhone districts of Savannakhet province. The sociodemographic information includes information related to the respondents as well as to their households, namely gender, marital status, age, level of education, household size, income level of household, existence of chronic condition within their households, and the respondents’ occupation.

**Table 2:** Sociodemographic characteristics and hospital admission (IPD)^a^

Sociodemographic of respondents/households	Hospital admission (IPD)			Pearson χ^2^
	No	Yes	Total	*P* value
Gender of respondents				
Male	131 (55.5%)	59 (55.7%)	190 (55.6%)	0.979
Female	105 (44.5%)	47 (44.3%)	152 (44.4%)	
Marital status				
Single	52 (22%)	30 (28.3%)	82 (24%)	0.209
Married	184 (78%)	76 (71.7%)	260 (76%)	
Age				
18–35	62 (26.3%)	21 (19.8%)	83 (24.3%)	0.431
36–49	74 (31.4%)	37 (34.9%)	111 (32.5%)	
50 or above	100 (42.4%)	48 (45.3%)	148 (43.3%)	
Level of education				
Never attended school	58 (24.6%)	25 (23.6%)	83 (24.3%)	0.959
Primary school	86 (36.4%)	38 (35.8%)	124 (36.3%)	
Lower secondary school or higher	92 (39%)	43 (40.6%)	135 (39.5%)	
Size of household				
1–4 people (small)	119 (50.4%)	54 (50.9%)	173 (50.6%)	0.929
5 people or more (large)	117 (49.6%)	52 (49.1%)	169 (49.4%)	
Level of income				
Less than 1 million LAK (US$120)	80 (33.9%)	30 (28.3%)	110 (32.2%)	0.563
1 million (US$120) to 2.5 million (US$300)	67 (28.4%)	31 (29.2%)	98 (28.7%)	
2.5 million (US$300) or more	89 (37.7%)	45 (42.5%)	134 (39.2%)	
Chronic condition				
No	206 (87.3%)	49 (46.2%)	255 (74.6%)	0.000^b^
Yes	30 (12.7%)	57 (53.8%)	87 (25.4%)	
Occupation				
Casual worker	73 (30.9%)	30 (28.3%)	103 (30.1%)	0.064
Farmer	66 (28%)	20 (18.9%)	86 (25.1%)	
Street vendor	45 (19.1%)	33 (31.1%)	78 (22.8%)	
Labourer	52 (22%)	23 (21.7%)	75 (21.9%)	

*IPD* inpatient department

^a^Based on the 2018 data collection in Kaysone Phomvihane district and Champhone district of Savannakhet province

^b^Statistically significant at 95% confidence interval

**Table 3:** Sociodemographic characteristics and catastrophic health expenditure^a^

Sociodemographic of respondents/households	Catastrophic health expenditure			Pearson χ^2^
	No	Yes	Total	*P* value
Gender of respondents				
Male	41 (55.4%)	18 (56.3%)	59 (55.7%)	0.936
Female	33 (44.6%)	14 (43.8%)	47 (44.3%)	
Marital status				
Single	24 (32.4%)	6 (18.8%)	30 (28.3%)	0.151
Married	50 (67.6%)	26 (81.8%)	76 (71.7%)	
Age				
18–35	17 (23%)	4 (12.5%)	21 (19.8%)	0.461
36–49	25 (33.8%)	12 (37.5%)	37 (34.9%)	
50 or above	32 (43.2%)	16 (50%)	48 (45.3%)	
Level of education				
Never attended school	19 (25.7%)	6 (18.8%)	25 (23.6%)	0.421
Primary school	28 (37.8%)	10 (31.3%)	38 (35.8%)	
Lower secondary school or higher	27 (36.5%)	16 (50%)	43 (40.6%)	
Size of household				
1–4 people (small)	44 (59.5%)	10 (31.3%)	54 (50.9%)	0.008^b^
5 people or more (large)	30 (40.5%)	22 (68.8%)	52 (49.1%)	
Level of income				
Less than 1 million LAK (US$120)	17 (23%)	13 (40.6%)	30 (28.3%)	0.046^b^
1 million (US$120) to 2.5 million (US$300)	20 (27%)	11 (34.4%)	31 (29.2%)	
2.5 million (US$300) or more	37 (50%)	8 (25%)	45 (42.5%)	
Chronic condition				
No	34 (45.9%)	15 (46.9%)	49 (46.2%)	0.930
Yes	40 (54.1%)	17 (53.1%)	57 (53.8%)	
Occupation				
Casual worker	20 (27.0%)	10 (31.3%)	30 (28.3%)	0.879
Farmer	13 (17.6%)	7 (21.9%)	20 (18.9%)	
Street vendor	24 (32.4%)	9 (28.1%)	33 (31.1%)	
Labourer	17 (23%)	6 (18.8%)	23 (21.7%)	

^a^Based on the 2018 data collection in Kaysone Phomvihane district and Champhone district of Savannakhet province

^b^Statistically significant at 95% confidence interval

With regards to the descriptive statistics between the sociodemographic characteristics and hospital admission (Table 2), in terms of Pearson χ^2^ value, only the existence of a chronic condition within the household was statistically significant. It was indicated that, among 106 hospital admissions, 57 households (or 53.8%) had at least one member suffering from a chronic condition, with the significant Pearson χ^2^ value of 0.000, proving that the relationship between existence of chronic condition and hospital admission (IPD) were not independent.

Table 3 describes the relationship between the sociodemographic and catastrophic health expenditure, referring to the case where a household must reduce its basic expenses, over a specific period of time, to make it possible to afford health services. Specifically, it should be recalled that catastrophic health expenditure is a situation where healthcare expenditure is greater, or equal to, 40% of the capacity to pay. Capacity to pay is defined as non-subsistence effective income, of which subsistence spending is equal to one dollar, per day, per person according to WHO [29]. Among 106 households reported to be using the IPD health services, 32 households were considered as suffering from financial catastrophe; the result shows that larger-sized households (more than five people) have a larger portion of catastrophic health expenditure, at 68.8%, with a Pearson χ^2^ *P* value of 0.008. In addition, households within the lowest income quantile (less than 1 million LAK or approximately 120 USD) have a higher share, at 40.6%, in comparison to other income quantiles in terms of catastrophic health expenditure, with a Pearson χ^2^ *P* value of 0.046. Significant Pearson χ^2^ values mean that there were relationships between the size of households and catastrophic health expenditure as well as the level of income and catastrophic health expenditure.

The information in Table 4 describes the probability of hospitalisation under the CBHI scheme during two different time periods (2013 and 2016) and under NHI (in 2018). The probability of hospitalisation is used as proxy data to observe the accessibility of hospitalisation. Under the CBHI scheme, a study conducted in 2013 found that only the existence of a chronic condition had a 1.786 higher probability of hospital admission when compared to a household without any chronic condition [26]. An identical study design, conducted in 2016, indicated that a chronic condition within a household had a significant impact on hospital admission; existence of a chronic condition within a household led to a 2.326 higher probability of hospital admission in comparison to households without a chronic condition [27]. With regards to insurance status, insured households had a 1.803 higher probability of hospital admission when compared to uninsured households. That is to say, the study in 2016 found that CBHI was able to improve accessibility of health service utilisation.

**Table 4:** Probability of hospitalisation under Community-Based Health Insurance (CBHI) and National Health Insurance (NHI) schemes

Independent variable (Andersen’s Behavioral Model)	Binary logistic regression model 1: Probability of hospitalisation									
	CBHI								NHI	
	2013 (controlling insurance status)		2013		2016 (controlling insurance status)		2016		2018	
	Nagelkerke R^2^ 0.076		Nagelkerke R^2^ 0.069		Nagelkerke R^2^ 0.097		Nagelkerke R^2^ 0.076		Nagelkerke R^2^ 0.248	
	OR	*P* value	OR	*P* value	OR	*P* value	OR	*P* value	OR	*P* value
Predisposing factors										
Gender										
Male										
Female	1.472	0.200	1.451	0.151	0.698	0.157	0.712	0.160	0.882	0.815
Age										
18–35										
36–49	0.660	0.312	0.674	0.345	0.887	0.681	0.965	0.798	1.524	0.357
50 or above	0.835	0.673	0.800	0.712	0.797	0.485	0.900	0.598	2	0.648
Marital status										
Single										
Married	0.913	0.863	0.901	0.821	1.260	0.348	1.430	0.569	3.610	0.050*
Educational status										
Never attended school										
Primary school	0.643	0.310	0.679	0.387	0.707	0.258	0.876	0.468	1.371	0.150
Lower secondary school or higher	1.181	0.705	1.235	0.754	0.993	0.983	1.076	0.900	3.205	0.188
Size of household										
Small (1–4 people)										
Large (more than 5 people)	0.907	0.761	0.902	0.752	1.283	0.324	1.376	0.453	5.128	0.02*
Enabling factors										
Income level										
Less than 1 million LAK (US$120)										
1 million (US$120) to 2.5 million LAK (US$300)	0.612	0.150	0.643	0.170	0.664	0.194	0.743	0.348	0.516	0.037*
2.5 million LAK (US$300) or more	0.483	0.088	0.421	0.076	1.065	0.834	1.327	0.898	0.135	0.08
Insurance status										
No – Uninsured										
Yes – Insured	1.455	0.231			1.803	0.021*				
Need factors										
Chronic condition										
No										
Yes	1.786	0.057*	1.796	0.065	2.326	0.003*	2.459	0.005*	0.960	0.935

* Statistically significant at 95% confidence interval

Bearing in mind that the NHI was introduced in 2017 as a pilot project in Savannakhet province, a binary logistic regression model was once again used to evaluate its impact. Since there is no enrolment required for the NHI, any group of the population, other than government officials (under the State Authority for Social Security), are eligible to use both OPD and IPD services. As a result, there is no ‘insurance status’ in the binary logistic regression model. The results indicated that married respondents, large households and the level of income had a significant impact on hospital admission (accessibility to health service utilisation). Specifically, married households have 3.610 times higher chances of hospital admission than non-married households. At the odds ratio (OR) of 5.128, large households with more than five people have 5.128 higher probability than those with no admission and their small household counterparts. In terms of income level, given that the OR is 0.516 for the medium-income household variable, the medium-income households have a 0.516 times higher chance of being admitted than their low-income counterparts. In other words, the low-income households have 1.937 (inverted OR 1/0.516) times higher chance of being admitted than medium-income households. The comparison of three studies found that, under the NHI, the socioeconomic characteristics that were statistically significant were married respondents, large households, and households with a monthly income of between 1 and 2.5 million LAK (120 – 300USD); whereas no socioeconomic characteristics and only one sociodemographic variable were statistically significant in 2013 and 2016, respectively [26, 27].

To be comparable with a case study of the NHI, logistic regression models were used to analyse the probability of hospitalisation in 2013 and 2016, again without an insurance status (with the identical data set). The results yielded a very similar context to the original logistic models, in which the binary logistic regression on probability of health service utilisation under the CBHI scheme in 2013 (without insurance status included in the model) found that households with a chronic condition have a 1.796 times higher probability of using health services compared to healthy households without the existence of a chronic condition. However, the *P* value was not statistically significant at 95% confidence interval. In 2016, similar logistic regression showed the chronic condition as still being the most important factor behind health service utilisation. Pseudo R^2^ (Nagelkerke R^2^) values of each binary logistic regression model are also presented in Table 4 to estimate the goodness-of-fit of each model. The Nalkerke R^2^ under NHI (column ‘2018’) had the highest value of 0.248, indicating that independent variables together account for 24.8% of the reasons for accessing health services. Another important observation is that, under the CBHI scheme (2013 and 2016), controlling insurance status models provided a better goodness-of-fit with relatively higher Nagelkerke R^2^.

Catastrophic expenditure was estimated by comparing the yearly income and the amount of health service spent in the last 12 months, wherein households with health-related expenditures (medical and non-medical expenditure) of more than 40% of their income were categorised as in financial catastrophe. Table 5 compares the effectiveness of financial protection against catastrophic health expenditure between the two studies under the CBHI scheme and the NHI. In 2013, under the CBHI scheme, the result shows that only household income levels were statistically significant at a 95% confidence interval. According to Table 5, the middle-income households’ OR was 0.049, while that of high-income households was 0.34. The odds of having catastrophic expenditure over not having catastrophic expenditure for middle-income households (1 million or 120 USD to 2.5 million or 300 USD) were 0.049 compared to low-income households (less than 1 million LAK or 120 USD), whereas those of high-income households (more than 2.5 million LAK or 300 USD) were 0.34 compared to low-income households. Additionally, the model also found that a household with at least one household member suffering from a chronic condition had a 4.306 higher probability of incurring catastrophic health expenditure when compared to a household without a chronic condition. A second study, conducted in 2016, found similar results in terms of income level, in which the odds of having catastrophic expenditure for middle-income households and high-income households were 0.030 and 0.012 compared to low-income households, respectively. After the introduction of the NHI as a pilot project in 2017, a similar binary logistic regression model found that only the existence of a chronic condition within a household was a factor of statistical significance, at a 95% confidence interval. Statistical analysis proved that the existence of a chronic condition within a household resulted in an 8.695 times higher probability of financial catastrophe, due to hospitalisation, in comparison to households with healthy members. Despite the *P* values of higher-income households not being statistically significant, the ORs (1.166 and 1.117) show that higher-income households have more possibility of suffering from financial catastrophe compared to lowest-income households. The availability of the NHI programme seems to encourage relatively well-off households to use health services because the lowest-income household may not be able to afford non-medical expenditures. On the other hand, the poorest income households are still reluctant to use health services since they worry about non-medical expenditure, including transportation costs, food expenditure of both patients and their companies (during hospitalisation), and accommodation expenditures (sometimes patients and their family have to stay in individual rooms, which is not covered by NHI due to the huge inflow of patients). As NHI is a new pilot programme, not much information about NHI policy has been distributed to poor people who sometimes prefer going to see local private clinics, visiting shamans and self-prescription. This statement is in tandem with the information provided in Table 2, illustrating that there are higher proportions of household income of more than 2.5 million LAK (300 USD) and 1 million (120 USD) to 2.5 million LAK (300 USD) in comparison to the poorest income quantile with less than 1 million LAK (120 USD). Similarly, for the case of financial protection, the logistic regression model without insurance status was analysed again for the data set under CBHI in 2013 and 2016. The logistic regression model predicting the probability of financial catastrophe (without insurance variable) yielded very similar results. In 2013, the highest-income quintile (more than 2.5 million LAK or 300 USD) and the middle-income quintile (1 million or 120 USD to 2.5 million or 300 USD) were 0.056 and 0.045 times when compared to low-income households, respectively. When comparing the Nagelkerke R^2^ values presented in Table 4, the binary logistic regression models predicting the probability of experiencing financial catastrophe had a better explanation. However, controlling insurance status under CBHI schemes (in 2013 and 2016) provided better goodness-of-fit in comparison to binary logistic regression models without insurance status.

**Table 5:** Probability of having a financial catastrophe under the Community-Based Health Insurance (CBHI) and National Health Insurance (NHI) schemes

Independent variable (Andersen’s Behavioral Model)	Binary logistic regression model 2: catastrophic expenditure (of inpatient department)									
	CBHI								NHI	
	2013 (controlling insurance status)		2013		2016 (controlling insurance status)		2016		2018	
	Nagelkerke R^2^ 0.594		Nagelkerke R^2^ 0.583		Nagelkerke R^2^ 0.635		Nagelkerke R^2^ 0.415		Nagelkerke R^2^ 0.301	
	OR	*P* value	OR	*P* value	OR	*P* value	OR	*P* value	OR	*P* value
Predisposing factors										
Gender										
Male										
Female	1.990	0.368	2.012	0.453	0.838	0.804	0.743	0.795	0.662	0.601
Age										
18–35										
36–49	0.314	0.304	0.543	0.651	0.287	0.146	0.362	0.154	1.223	0.211
50 or above	0.140	0.089	0.156	0.094	0.039	0.007	0.123	0.078	0.803	0.457
Marital status										
Single										
Married	1.648	0.687	1.431	0.541	1.572	0.524	1.659	0.573	0.643	0.144
Educational status										
Never attended school										
Primary school	0.779	0.818	0.631	0.756	0.245	0.094	0.346	0.097	0.943	0.505
Lower secondary school or higher	0.114	0.045	0.124	0.056	0.522	0.472	0.542	0.871	1.156	0.792
Size of household										
Small (1–4 people)										
Large (more than 5 people)	1.978	0.364	2.142	0.534	1.026	0.970	1.042	0.879	0.946	0.836
Enabling factors										
Income level										
Less than 1 million LAK (US$120)										
1 million (US$120) to 2.5 million LAK (US$300)	0.049	0.000*	0.056	0.001*	0.030	0.000*	0.056	0.002*	1.166	0.894
2.5 million LAK (US$300) or more	0.034	0.019*	0.04	0.003*	0.012	0.000*	0.045	0.000*	1.117	0.900
Insurance status										
No – uninsured										
Yes – Insured	0.426	0.277			0.037	0.000*				
Need factors										
Chronic condition										
No										
Yes	4.306	0.067	6.102	0.083	0.622	0.568	0.75	0.780	8.695	0.000*

* Statistically significant at 95% confidence interval

## Discussion

It should be remembered that the main objective of this study is to compare the outcomes of the voluntary CBHI scheme and the newly promoted NHI in terms of accessibility of health service utilisation and financial protection against catastrophic health expenditure. Under the CBHI scheme, members are required to pay membership fees (also known as the contribution rate). Health service utilisation, or accessibility to health services, were strongly affected by the existence of a chronic condition within the household; a regular situation arose in the health insurance scheme, known as adverse selection, where people who are prone to suffering from health problems are more likely to acquire health insurance, since the insurance scheme cannot discriminate against this group of the population, which possibly forces them, by law, as well as other constraints [30]. However, the CBHI scheme is able to ease or enhance health service utilisation for the insured households. This outcome is in tandem with a study in Mexico, which found that the voluntary health insurance scheme (known as Seguro Popular) is effective in providing protection against financial hardship [31].

After the introduction of the NHI, there has been an improvement in terms of accessibility to health service utilisation as the NHI is able to significantly increase health service utilisation for the poorest income quantile households (earning less than 1 million LAK or 120 USD/month), whereas under the preceding CBHI scheme, the increase in accessibility was not statistically significant at a 95% confidence interval. A possible reason supporting this statement is that, under the NHI, poor households do not have to pay monthly or annual contributions to secure their free health service utilisation. Therefore, the NHI offers better health service distribution to low-income households. Consequently, people from within any income quintile could equally go to a public hospital without bearing the full health expenditure. The introduction of NHI subsequently creates more health service utilisation. Without improvement and expansion of the hospitals’ capacity and human resources, the public hospitals will become very crowded and overloaded with patients. As a result, most of the patients in the upper income quintiles prefer to travel to neighbouring countries (in the belief of receiving better health services) for their treatment. Additionally, the regression model also found that, under NHI, married respondents and large households were more likely to have better accessibility to health services. The outcome of a previous study, conducted in the Philippines, found that poverty incidence worsens as households grow; the improved accessibility of health services for large households refers to the NHI enabling an increase in accessibility for the larger or lower-income households [32].

Regarding the assumption that there is an improvement in accessibility under the NHI, this could be due to the fact that people could have more medical knowledge over time and thus increase their number of hospital visits or people could have become exposed to larger risk factors and non-communicable diseases over time, resulting in higher hospitalisation rates. However, this study was conducted not long after the introduction of the NHI (in replacement of the CBHI), targeting an identical group of the population. In theory, both CBHI and NHI aim to improve accessibility to all population groups, but in practice most of the people enrolling in the CBHI scheme, or using NHI, were low- to middle-income households such that, within a short period of time, those would not really show an improvement in terms of their medical knowledge. As there has been no outbreak or sudden increase in occurrences of disease, the increase in the probability of hospitalisation should not be affected by those factors. This statement means that the NHI is effectively easier to access for the general population, compared to its predecessor.

In terms of financial protection against catastrophic health expenditure, insured households were significantly protected by the CBHI scheme. However, the poorest income quantile still retained the highest probability of suffering catastrophic heath expenditure; this condition is very similar to that of any developing country, other than South Africa, where primary healthcare is provided free of charge to all citizens [33]. Under the NHI scheme, an existing chronic condition within a household is the significant factor that leads to a catastrophic health situation. This result is comparable to that of a study previously conducted in China, which revealed that IPD health service utilisation was more likely to lead to suffering with catastrophic heath expenditure; this indicates that the health financing system in China is unable to lower the possibility of catastrophic spending, nor relieve the financial burden of households with a chronic condition [34, 35].

The context of health financing in the Lao People’s Democratic Republic is very similar to that of a study on equity in financing and health service utilisation in Ghana, South Africa and Tanzania that found three main constraints related to availability, affordability and acceptability [36]. Primarily, health service utilisation in the Lao People’s Democratic Republic encounters serious issues regarding the availability constraint; for instance, transportation to health facilities in the Lao People’s Democratic Republic is very limited and road conditions are not convenient, proves time consuming for patients to be delivered to their closest public health facilities. Additionally, a large number of patients from other districts are transferred to a referral hospital (Savannakhet provincial hospital) in Kaysone Phomvihane district, resulting in patient overload. The most notable issue is that chronic kidney disease patients are not able to wait for haemodialysis treatment in Savannakhet provincial hospital (due to the limited number of haemodialysis machines and qualified health practitioners). The number of health personnel per 1000 population in the Lao People’s Democratic Republic is very limited, with a doctor to population ratio and a nurse to population ratio of 0.019 and 0.082, respectively, whereas in a neighbouring country such as Thailand, the doctor to population ratio is 0.47 and the nurse to population ratio is 2.08 [37–39].

Consequently, a large number of patients opt to use the more expensive health services in Mukdahan province, Thailand. Furthermore, the unavailability of essential drugs, skilled health practitioners and diagnostic equipment are also important issues; this has created a situation where, regardless of their insurance status (even patients who are covered by health insurance), some decide to undertake OOP expenditure to seek treatment in foreign hospitals. In terms of affordability constraints, transportation costs to access public health facilities remain very high, especially from the rural and mountainous areas; consequently, this leads to an increase in their non-medical expenditure. Drugs are often only available from private pharmacies, at a higher cost than from government-owned drug stores. According to the NHI policy, patients are expected to pay 25% (as a co-payment) for surgery costs of over 5 million LAK (600 USD), causing financial catastrophe for poor households.

During 2013–2018, there were improvements in terms of accessibility and financial protection. Accessibility to health services have been significantly improved for poor households under NHI compared to the preceding CBHI scheme. In terms of financial protection, the model found that income levels do not have any significant impact on the possibility of experiencing financial catastrophe. In other words, NHI eases the financial issues for all income quantiles and lowers the cost of health service utilisation in general. This situation is very similar to research outcomes from India and South Africa that revealed people covered by private insurance schemes may have been encouraged to use special healthcare that resulted in higher co-payments, which increase the probability of suffering from catastrophic health expenditure [40]. Regarding the acceptability constraints, this study found that patients do not have real confidence in the quality of the health services provided by their local health provider. As in other developing countries, a large number of patients in the Lao People’s Democratic Republic complain about the poor attitudes of the health practitioners, which discourages the use of the health service locally. This lack of confidence in the health system has been viewed both by locals and foreigners who are residing in the Lao People’s Democratic Republic. As an example, the Australian embassy provides medical advice through its official website claiming that the medical facilities outside Vientiane capital are limited and totally inadequate in rural areas (especial in the northern parts of the country). People with medical issues should consider, in advance, when travelling to remote areas that there is no health practitioner and suitable health facilities to treat serious health conditions. Despite the availability of some western-trained physicians, health facilities and equipment are not properly maintained. The Australian embassy in the Lao People’s Democratic Republic recommends to their people that those who need health services should go to hospitals in Udon Thani province, Thailand [41]. Consequently, regardless of the financial protection, most of the upper-income households prefer to use the health services in neighbouring countries to ensure their satisfaction.

## Conclusion

OOP expenditure remains the major means of health expenditure in developing countries, limiting households in accessing health services and possibly creating a financial catastrophe [29]. To increase accessibility and minimise health expenditure, in 2002, the government of the Lao People’s Democratic Republic established a voluntary scheme targeting the non-poor and independent workers (not working under the government or a registered private company).

Previous studies found that the voluntary health insurance scheme, CBHI slowly improved accessibility to quality health services provided by public health practitioners and offered some financial protection against catastrophic health expenditure. Despite requiring only small levels of contribution, the scheme suffered from low enrolment and a high dropout rate. As there was no gate-keeping mechanism to prevent members dropping out of the scheme (after receiving use of the CBHI’s benefit package), this spontaneously lowered the CBHI’s risk pooling level. Without any restriction, former CBHI members were free to re-enrol in the same scheme ‘sometime in the future’, which could be following the expectation of a huge operation or other health service requirement. Therefore, the government decided to pilot the NHI in many provinces, combining numbers of existing schemes as a stepping stone towards a universal health insurance system. Unlike previous health financing schemes, patients are expected to pay a flat contribution rate at the time of using the health service and a co-payment of 25% for medical expenditure higher than 5000,000 LAK (600 USD).

The logistic regression model found that the NHI significantly enhances accessibility to healthcare for low-income households (income of less 1 million LAK or 120 USD/month), improving health service distribution or accessibility for the various income levels in comparison to the CBHI coverage. In terms of financial protection, the model found that the socioeconomics categorised in predisposing and enabling characteristics were not statistically significant, meaning that the NHI had enhanced financial protection since its introduction. The only factor that was statistically significant was the existence of a chronic condition; this meant that, regardless of the hospitalisation cost (under the NHI coverage), the existence of a chronic condition is still considered as the important factor that significantly increases the probability of encountering catastrophic health expenditure. However, the NHI policy requires a dramatically high level of government subsidy; therefore, its long-term sustainability remains to be determined. To ensure sustainability in the long run, it is highly recommended that the government should improve financial management and expenditure systems in all levels of the health system. Additionally, the government must rely on evidence-based priority-setting to identify which of its limited resources should be developed and enhanced [9]. The findings in this study prove that the newly piloted NHI is able to promote both accessibility and financial protection. However, this does not mean that everyone who is sick and all people with health problems will be able to access health facilities without them being improved or by increasing the numbers of facilities, medical equipment and health personnel. Before fully implementing the NHI throughout the country, the government should improve the aforementioned factors in order to be capable of managing the large inflow of patients. Additionally, under the NHI, patients are responsible for co-payment, which may push them into a catastrophic condition and poverty. Regarding this issue, the policy related to the co-payment system should be revised, for example, to allow patients and their families to pay by instalments or provide a special co-payment rate for the very poor households. A possible limitation of this study could be its external validity and its small sample size, which many not fully represent the population group. Although self-reporting has been used in many studies [42], self-reporting on chronic conditions and other household characteristics were considered as a limitation of this study. This limitation was minimised by using a proper number for the sample size and reliable tools for implementing reliability and validity prior to the interview process.

## Data Availability

Please email to somdethb@rocketmail.com for: Information for respondents and informed consent. Questionnaire for respondents. SPSS data base.

## Abbreviations

CBHI: Community-Based Health Insurance
HEF: Health Equity Fund
IPD: inpatient department
LAK: Lao KIP
NHI: National Health Insurance
OOP: out-of-pocket
OPD: outpatient department
OR: odds ratio
RDF: Revolving Drug Fund
SSO: Social Security Organization
